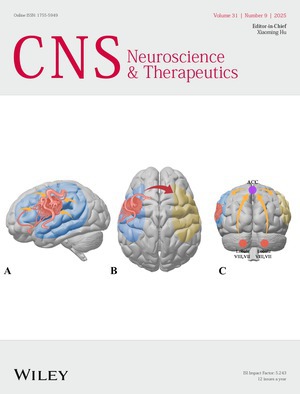# Front Cover

**DOI:** 10.1111/cns.70622

**Published:** 2025-09-22

## Abstract

The cover image is based on the article *Language Functional Connectivity Alterations During Resting State in Brain Arteriovenous Malformation Patients* by Xiaofeng Deng et al., https://doi.org/10.1111/cns.70602.